# A graph-theoretical representation of multiphoton resonance processes in superconducting quantum circuits

**DOI:** 10.1038/srep37544

**Published:** 2016-11-21

**Authors:** Hossein Z. Jooya, Kamran Reihani, Shih-I Chu

**Affiliations:** 1 Department of Chemistry, University of Kansas, Lawrence, Kansas 66045, USA; 2 Department of Mathematics, Texas A&M University, College Station, Texas 77843, USA; 3 Center for Quantum Science and Engineering, Department of Physics, National Taiwan University, Taipei 10617, Taiwan

## Abstract

We propose a graph-theoretical formalism to study generic circuit quantum electrodynamics systems consisting of a two level qubit coupled with a single-mode resonator in arbitrary coupling strength regimes beyond rotating-wave approximation. We define colored-weighted graphs, and introduce different products between them to investigate the dynamics of superconducting qubits in transverse, longitudinal, and bidirectional coupling schemes. The intuitive and predictive picture provided by this method, and the simplicity of the mathematical construction, are demonstrated with some numerical studies of the multiphoton resonance processes and quantum interference phenomena for the superconducting qubit systems driven by intense ac fields.

Dressed atom formalism was developed in 1969 by Cohen-Tannoudji and Haroche^1^ to explain the behavior of atoms exposed to radio-frequency fields described in terms of photons^2^. In fact, the Floquet quasienergy diagram is equivalent to the fully quantized dressed-atom picture in the limit of strong fields^3^. Generalization of the Floquet theory for non-perturbative treatment of infinite-level systems, including both bound and continuum states, was first introduced by Chu and Reinhardt^4^ in 1977. Dressed superconducting qubits^5^,^6^, have been theoretically studied^7^, and experimentally demonstrated^8^,^9^. For strongly driven superconducting qubits, the Floquet formalism can describe the ac Stark level shift and power broadening for multiphoton resonance processes, which appear beyond rotating-wave approximation (RWA)^10^. Also, the RWA is not applicable in the ultrastrong-coupling (USC) regime^11^,^12^. This new regime of cavity quantum electrodynamics (QED), where the coupling rate becomes an appreciable fraction of the unperturbed frequency of the bare systems, has been experimentally reached in a variety of solid state systems^13^,^14^,^15^,^16^. In RWA the excitation-number-nonconserving processes or virtual transitions have been excluded in calculations. Therefore the Jaynes-Cummings model cannot describe higher order resonant transitions via intermediate states connected by counter-rotating terms^17^,^18^.

The main purpose of this paper is to provide a generalized and systematic graph theoretical approach^19^,^20^, motivated by dressed states picture of Floquet theory, to analyze a generic circuit QED system consisting of a two-level qubit coupled to a single-mode resonator in any arbitrary coupling strength regime. We will demonstrate the results from the small normalized coupling rate to deep strong coupling (DSC) regime, where the coupling strength is compatible or larger than the oscillator frequency^21^. By considering the counter-rotating terms, this graph-theoretical method will allow us to represent the virtual transitions, which are proven to be essential when the number of excitations in the cavity-emitter system is no longer conserved^13^.

Here, the proposed graph theoretical construction of the interacting quantum system should not be connected to a totally different concept of quantum graph^22^. Quantum graphs are usually introduced either through a differential operator acting on the functions defined on the edges of a graph or through directly specifying the scattering matrices at the vertices of the graphs^23^. An important relevance of the proposed graph product scheme is its natural connection to the so-called *Floquet Hilbert space*^24^. One can assign a natural Hilbert space to each graph, *G*, by considering all square summable, complex-valued functions on the vertex set *V*_*G*_ = *V*(*G*) Such Hilbert space is usually denoted by 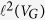. If *G* is 2-colored, then the coloring operator C_G_ defines a grading on the Hilbert space 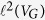, which becomes a *graded Hilbert space* (also called a *super Hilbert space*). The Floquet Hilbert space is defined as the Hilbert space tensor product


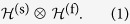


here 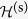 is the spin Hilbert space, and 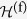 is the Fourier Hilbert space. The spin Hilbert space for a two-level system is 2-dimensional with basis 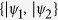, and the Fourier Hilbert space is infinite dimensional with basis 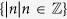
24,25. Consequently, we can naturally identify 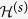 with 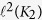 and 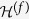 with 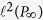 with 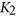 and 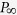 denoting the two node complete graph and infinite path graph, respectively. The dressed states


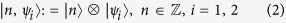


form a basis for the Floquet Hilbert space, and correspond to the vertices of any type of product between *K*_2_ and *P*_ ∞_. Moreover, when *K*_2_ is colored, the spin Hilbert space will be graded by the coloring matrix 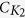, hence the resulting Floquet Hilbert space will be graded by considering the graded tensor product,


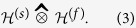


The picture provided by Floquet states are closely similar to the *n-*photon, or Fock states. Fock states are of special importance because quantum behavior in an oscillator is most obvious in these states^26^,^27^. They are frequently used in theoretical calculations, and form the basis of quantum computation and communications^28^. To see the resemblance between these pictures, we introduce the Hamiltonian for the quantum system as *H*_*Q*_ = *H*_*a*_ + *H*_*f*_ + *H*_*i*_, where *H*_*a*_ is the Hamiltonian of the atom, *H*_*f*_ is the Hamiltonian of the field, and *H*_*i*_ is the interaction Hamiltonian^21^,^29^. There are only two differences. In *H*_*Q*_, the index *n* runs from zero to infinity, but here it runs from −∞ to ∞. Also the off-diagonal elements of *H*_*Q*_ depend on *n* (e.g. proportional to 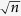 if *H*_*i*_ is proportional to the annihilation operator), whereas those of our matrices do not ref. 29.

## Mathematical Construction and Theoretical Details

Throughout this paper, the graphs are directed in general, but they don’t have loops (i.e., edges that connect a vertex to itself). The reported graphs are all weighted. By a weighted graph 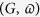, we mean a directed graph *G* with a countable vertex set *V(G)* and edge set 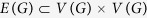 together with the additional labeling 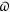 of the vertices and edges by complex numbers (called weights)^30^.

## Decomposition of a Weighted Graph

Let (*v*_*i*_) be a certain enumeration of the vertices of *G*. Denote the edge weight assigned to the directed edge (*v*_*i*_, *v*_*j*_) by 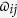 and the vertex weight assigned to *v*_*i*_ by 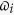. We make the convention that 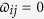 if and only if 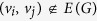 This defines the weighted adjacency matrix 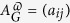 by^30^,^31^,


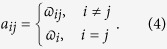


The weighted adjacency matrix is Hermitian if and only if all the vertex weights are real and the edge weights satisfy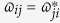. We are mainly interested in Hermitian adjacency matrices that correspond to quantum observables, chief among them being the Hamiltonian of our quantum systems. When the adjacency matrix is real and symmetric, we will simply consider an undirected graph with at most one edge between any two vertices 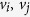 weighted by 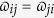. In particular, for an unweighted graph we can assume that 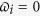 for all vertices, but any nonzero edge weight 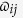 is equal to 1. Such a weight 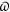 is said to be trivial, which obviously gives rise to the (unweighted) adjacency matrix of the graph. There is a one-to-one correspondence between weighted graphs and weighted adjacency matrices, so one can define a weighted graph given a symmetric matrix as its adjacency matrix^31^.

Each weighted adjacency matrix 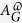 can be decomposed into the sum of a diagonal matrix 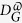 encoding the vertex weights and an off-diagonal matrix 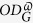 encoding the edge weights. In other words, we write 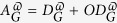, where 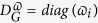. We call this the *weight decomposition* of 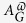. In particular, if 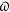 is trivial, 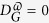 and 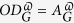.

## Colored Weighted Graphs and Their Products

Coloring vertices (or edges) of a graph is another method of labeling graphs. In this paper, we only consider the simplest case of vertex 2-coloring, namely, when all vertices are assigned by either of the two certain colors, and edges remain uncolored. A 2-coloring is said to be *proper* when every edge in the graph connects vertices with different colors, in which case the graph is called *bipartite*. Although properness is usually very useful in applications, we do not assume it for our graphs, unless it becomes a necessity. Suppose 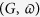 is a weighted graph with a certain 2-coloring of its vertices, say, blue and red. The information about coloring of vertices can be stored in a diagonal matrix 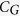 (coloring matrix) denoted by


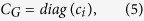


where *c*_*i*_ = 1 if the vertex *v*_*i*_ is blue, and *c*_*i*_ = −1 if the vertex *v*_*i*_ is red. Based on these definitions, a colored weighted graph is a triple 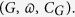 If the graph is uncolored, we simply assume that the coloring matrix *C*_*G*_ is the identity matrix. Let 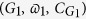 and 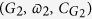 be two colored weighted graphs. Our goal is to define the colored weighted version of the well-known products (such as direct, Cartesian, and strong) between the two graphs. Let 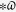 denote any such weighted product, e.g., 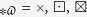 for direct, Cartesian, and strong product, respectively^30^. In all these cases, 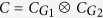 defines a coloring on the vertex set of the product graph. Here, the notation ⊗ stands for the Kronecker product of matrices^31^.

We now define the adjacency matrix of the *colored weighted direct product* as,


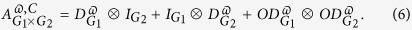


where 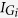 is the identity matrix of the size of graph *G*_*i*_.

The adjacency matrix of the *colored weighted Cartesian product* is introduced as,


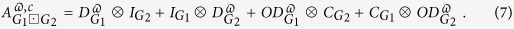


And finally, we define the adjacency matrix of the *colored weighted strong product* as,


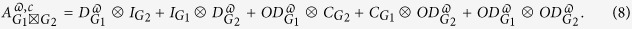


Eq. (8) implies that


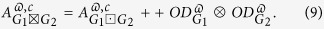


## Results and Discussion

Although the method can be applied to any number of multilevel systems, here, we limit our construction to a single two-level case. We model the two-level system as a two-dimensional spin Hilbert space 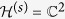 with orthonormal basis of 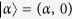, and 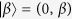, where *α* and *β* are real valued energies of the two states. Let *K*_2_ be a colored weighted graph with the weighted adjacency matrix


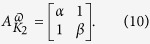


and with a vertex coloring defined as follows: the vertex weighted by *α* is red (so −1) and the vertex weighted by *β* is blue (so +1). We consider the interaction of this two-level system with a single mode oscillator. In general, the oscillating interaction connecting the states of the two-level system, *ε*(*t*), can be expanded with the Fourier components 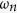
10,29,


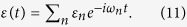


where 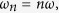 and 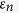 is the field amplitude corresponding to 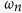. We model such discretized oscillating external field by a path graph, *P*_∞_. The vertices are assigned with the weights of 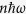, where 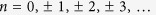.

The significance of our definition of colored-weighted graph products will be demonstrated by constructing the time-invariant Floquet Hamiltonian of a variety of QED systems. Without loss of generality, we consider a system consisting of a two level qubit coupled to a single-mode resonator in any arbitrary coupling strength regime. In the following we will show three case scenarios where the Floquet Hamiltonian *H*_*F*_ in an appropriate basis for the different physical models can be obtained as the weighted adjacency matrix of 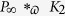, for either the direct, Cartesian, or strong product:


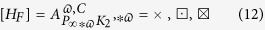


### Transverse coupling: Colored-weighted direct product

**Figure 1:**
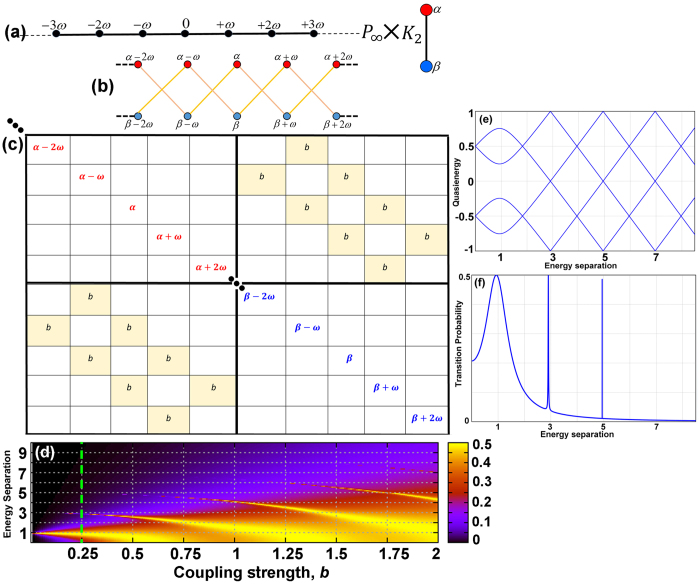
Transverse coupling. (**a**) Illustration of the *P*_∞_(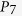
is presented) and *K*_2_ graphs before the direct production. *P*_∞_ models the discrete monochromatic laser field, and *K*_2_ represents the two-level system. (**b**,**c**) The schematic of the direct product graph, and its corresponding adjacency matrix are given, respectively. (**d**) The contour plot of the transition probability with respect to the coupling strength, *b*, and the energy separation between the two states. (**e**) Four branches of the quasienergies of the interacting system, and (**f** ) Transition probability as a function of energy separation for the two-level system driven by a single mode laser field with 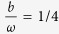, that is modeled by graph direct product *P*_∞_ × *K*_2_.

Natural atoms couple with electromagnetic fields at transverse mode due to the well-defined inversion symmetry of the potential energy^32^. Within the Bloch representation^33^, the time-dependent Hamiltonian of such two-level atom with transverse coupling is given by^29^,


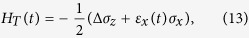


where 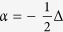, and 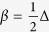. 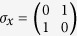, and 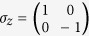 are Pauli matrices. Here, 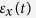 is the oscillating interaction connecting (through off-diagonal coupling) these states with a matrix element 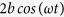, where *b* is the real-valued field amplitude, and *ω* is the main angular frequency. Atomic units are used throughout this paper. We set 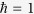. Following Eq. (11), for this case, all the Fourier coefficients, 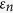, vanish, except for 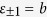. We assign equal weights of *b* to all the edges of the graph *P*_∞_. As mentioned before, we can now split the adjacency matrix of the oscillating field, which is modeled by the graph *P*_∞_, into the diagonal and off-diagonal terms^34^. In a 2 × 2 representative form,


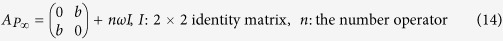


by definition, Eq. (6), the direct product *P*_∞_ × *K*_2_ gives the structure appeared in Fig. 1(b). The edges on this new graph product is weighted by *b*. The adjacency matrix, Fig. 1(c), for Eq. (6) generates the same structure as one obtains by applying the Floquet approach^29^.

After solving the eigenvalue problem for the colored weighted adjacency matrix of the direct product, the time-averaged transition probability between 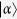 and 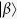 can be calculated as the probability to go from a single initial vertex on the product graph to a final vertex, summed over all the paths containing the intermediate vertices in the product graph. This can be numerically calculated as ref. 29.


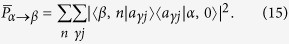


where *n* (and also *j*) is the Fourier index that runs over all the integers, *γ* = *α*, *β*, 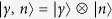 are the dressed states in the Floquet Hilbert space 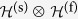, 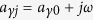 are the quasienergy eigenvalues of the product adjacency matrix, and 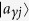 are the corresponding normalized eigenvectors. This is associated with the probability of finding the excited state of the qubit in the experiment. Figure 1(d) shows the contour map of the transition probability with respect to the coupling strength due to the external field, *b*, and the energy separation |*α* − *β*| between the two states of *K*_*2*_. Figure 1(e,f) present the qusienergy and the transition probability for the case when 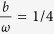. This result is in agreement with the results reported before^29^. As *b* increases, each resonance in Fig. 1(f) broadens and shifts toward smaller values of separation energy.

One can easily see the similarity between the schematic diagram of the direct product presented in Fig. 1(b) with the Hilbert space splitting in two unconnected subspaces or parity chains, *P* = +1, −1^21^. As schematically in Fig. 1(b), the direct product only allows odd-walks transitions in the graph. This is demonstrated, in Fig. 1(f), as the appearance of the transition peaks near the odd values of the separation energy. It can also be easily confirmed that these paths are only the passages responsible for the odd-multiphoton resonances. For example, removing all paths of length 3 from the product graph - by removing the corresponding edge weights from the adjacency matrix and the associated terms in Eq. (15) - eliminates the transition probability peak near the energy separation of 3 in Fig. 1(f).

### Longitudinal coupling: Colored-weighted Cartesian product

Since the potential energy for superconducting qubits can be tuned, the inversion symmetry for these artificial atoms can be broken and multiphoton processes can be observed^32^. The existence of the longitudinal coupling between superconducting qubits and applied magnetic fields were shown^35^, when the inversion symmetry of the potential energy of the superconducting qubit is broken. When a superconducting flux qubit is driven by a strong ac field, the time dependent Hamiltonian, which describes the longitudinal coupling is given by


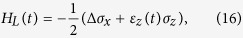


where 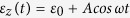
36. Here, the parameter Δ is the tunnel splitting and *ε*_0_ is the detuning energy proportional to the dc flux bias. *A* is the amplitude of the ac field that is parameterized in the energy unit and is proportional to the ac flux bias^37^. In this case, the adjacency matrix of the corresponding *K*_2_ graph which represents the two-level system is given by


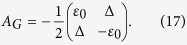


**Figure 2:**
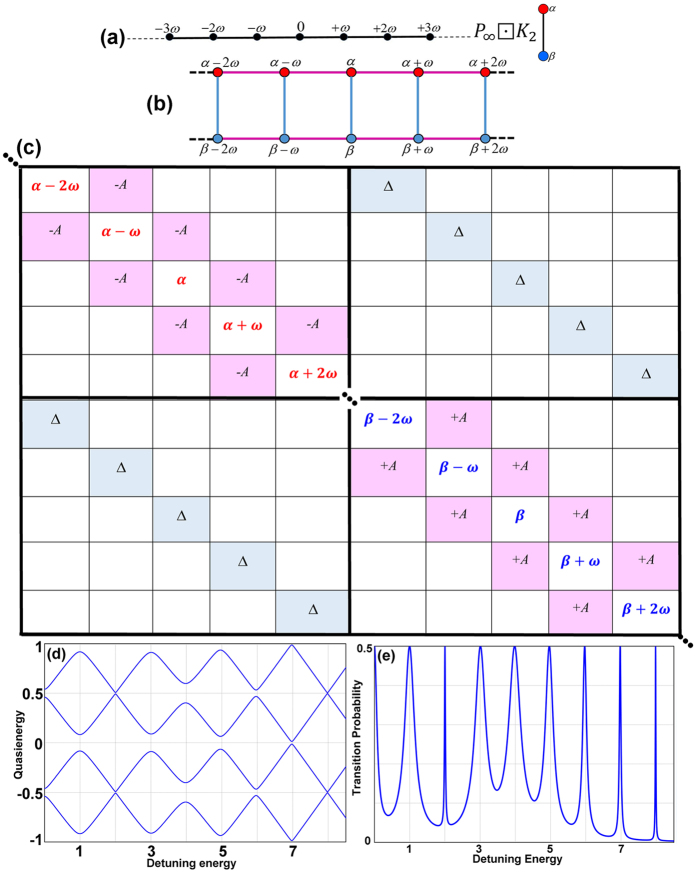
Longitudinal coupling. (**a**) Illustration of the 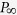 (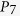 is presented) and *K*_2_ graphs before the Cartesian production. 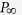 models the discrete monochromatic laser field, and *K*_2_ represents the two-level system. (**b**,**c**) The schematic of the Cartesian product graph, and its corresponding adjacency matrix are given respectively. (**d**) Four branches of the quasienergies of the interacting system, and (**e**) Transition probability as a function of detuning energy, 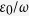, for the two-level system driven by a single mode laser field with 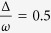 and 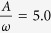, that is modeled by graph Cartesian product 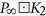.

The dynamics of such a system, as formulated by Eq. (16), can be modeled by the Cartesian product, 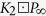, defined in Eq. (7). By this definition, the new product graph has the structure given in Fig. 2(b). The corresponding adjacency matrix is given in Fig. 2(c). Figure 2(d,e) present the qusienergy and the transition probability for this case as a function of the energy separation between the states of the two-level system with fixed parameters of 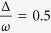 and 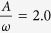, computed from the matrix presented in Fig. 2(c). The peaks in Fig. 2(e) at *nω* indicate the multiphoton resonance processes. This result is in agreement with the results reported before^10^. Due to the periodicity of the quasienergy, the quasienergy plot has repeating structure by *ω* with the avoided crossings between the lower and upper Floquet states in the vicinity of 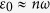 (*n* is a positive integer). At the avoided crossings the lower and upper Floquet states are strongly mixed and resonance transitions between 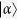 and 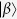 occur, as shown in the plot of time-averaged transition probability, Fig. 2(e). Also 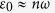 indicates that these transitions are multiphoton resonance processes.

**Figure 3:**
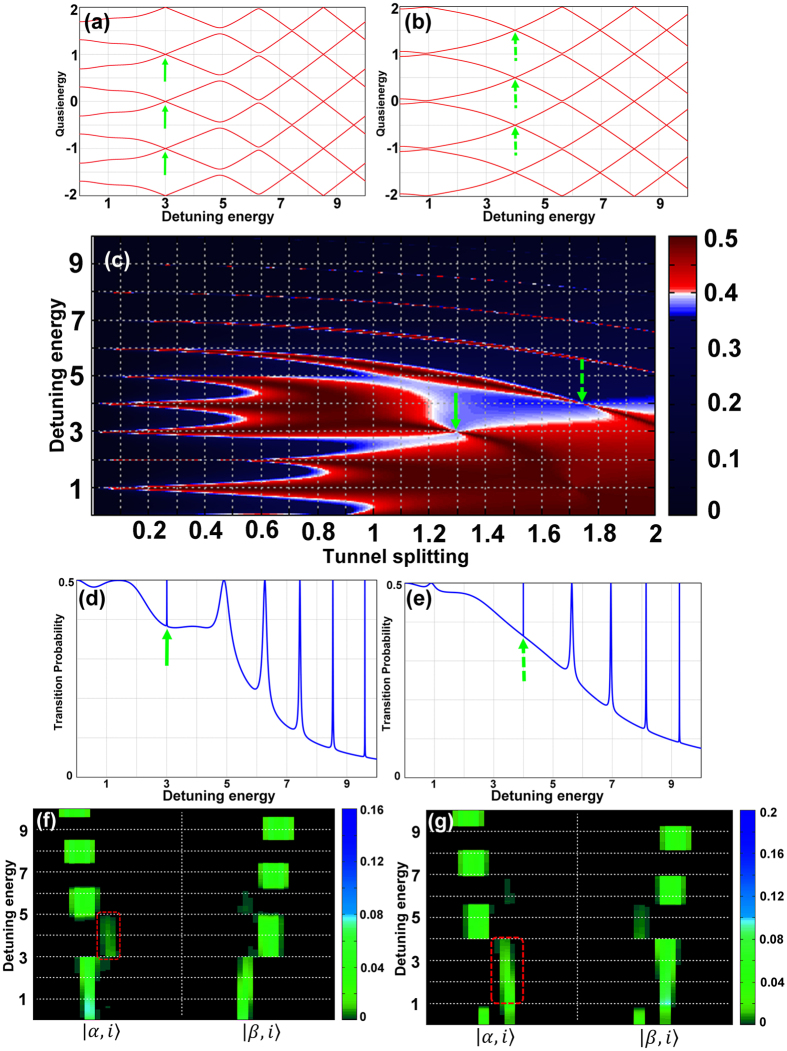
Multiphoton resonance suppression. The Appearance of multiphoton transition suppression at longitudinal coupling of a superconducting qubit with a single mode resonator. (**a**,**b**) The eight branches of the quasienergies of the system at the detuning energy of 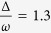 in (**a**), and 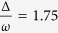 in (**b**). The narrowed avoided-crossings at the third and fourth photon resonances in (**a**,**b**) are shown by solid and dashed green arrows, respectively. (**c**) The contour plot of the transition probability with respect to the tunnel splitting parameter and the detuning energy between the two states. The existence of the third and fourth-photon resonance suppressions are indicated by the arrows. (**d**,**e**) The transition probabilities corresponding to (**a**,**b**). The narrowed peak in each case, indicated by solid and dashed green arrows, demonstrate the suppression of that particular resonance. (**f**,**g**) Population flipping between the *α* and *β* chains in the graph for the cases presented in (**d**,**e**), respectively. Indicated by the red-dashed boxes are the *α* -vertices with the population trapping.

The results presented in Fig. 3 show the appearance of three and four-photon resonance suppressions at longitudinal coupling between a superconducting qubit and a single mode resonator, when 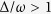. The transmission blockade of more than two photons was reported before, by tuning the frequency of the driving field to be equal to the sum of the Kerr nonlinearity and the cavity resonance frequency^16^,^38^,^39^. As indicated by the solid and dashed arrows in Fig. 3(d), the three-photon and four-photon transitions are dramatically suppressed at tunnel splitting 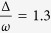 and 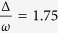, respectively. This nonlinear effect is emerged as the narrowed avoided crossings in the corresponding quasienergy plots, Fig. 2(a,b), and the slim transition probability distributions presented in Fig. 2(d,e). This effect is achieved by tuning the driving force to a suitably chosen frequency in the vicinity of an exact crossing of the corresponding two Floquet states. These multiphoton resonance suppressions can be explained by population trapping on the vertices connecting the final real states. For instance, in the case of three-photon resonance, these paths start from some initial state, eg. 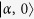, passes some virtual intermediate states that do not conserve energy^38^,^39^, and arrive at the real final state 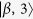. Figure 2(f,g) present the population flipping between the *α* and *β* rails on the ladder structure of the Cartesian graph product, Fig. 2(b). The red-dashed boxes indicate the *α* -vertices with the population trapping, which are responsible for the suppressed transition probability peaks in Fig. 2(d,e).

### Bidirectional coupling: Colored-weighted strong product

Superconducting qubits and the single mode field can have both transverse and longitudinal coupling^40^,^41^,^42^,^43^. The results presented so far have been focused on the use of linearly polarized (LP) laser fields. The use of elliptically polarized (EP) laser fields opens access to a number of strong-field processes that are either hindered or not present under the linear polarization^44^,^45^. The time-dependent Hamiltonian, which includes both the longitudinal and transverse coupling is given as,


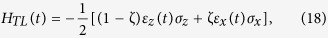


**Figure 4:**
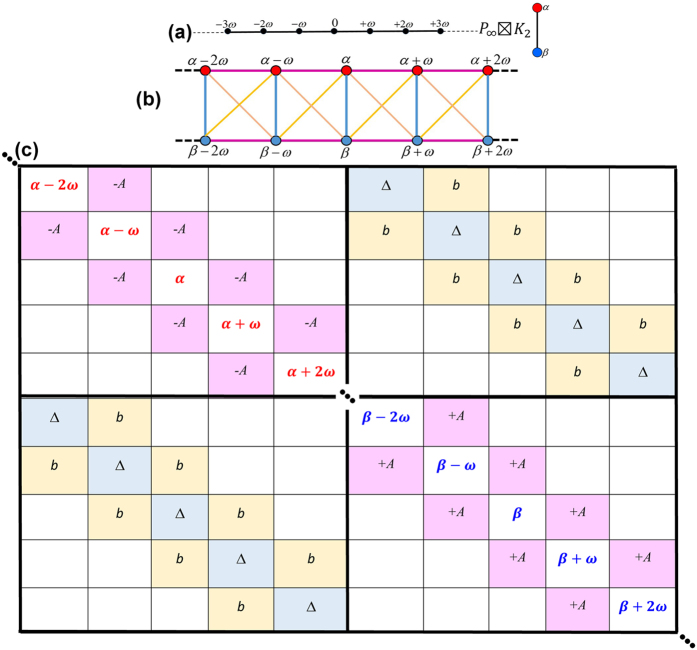
Bidirectional coupling. (**a**) Illustration of the 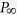 (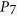 is presented) and *K*_2_ graphs before the strong production.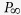 models the discrete monochromatic laser field, and *K*_2_ represents the two-level system. (**b**,**c**) The schematic of the strong product graph, and its corresponding adjacency matrix are given, respectively.

Where 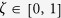 is the ellipticity parameter. As expected, this system can be modeled by combining the above two cases where the transverse and longitudinal couplings were investigated separately. The graph-theoretical approach of modeling this interaction is given in Eq. (8) as the strong product 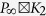 with the resulting structure presented in Fig. 4(b). The corresponding adjacency matrix for this product graph is given in Fig. 4(c).

**Figure 5:**
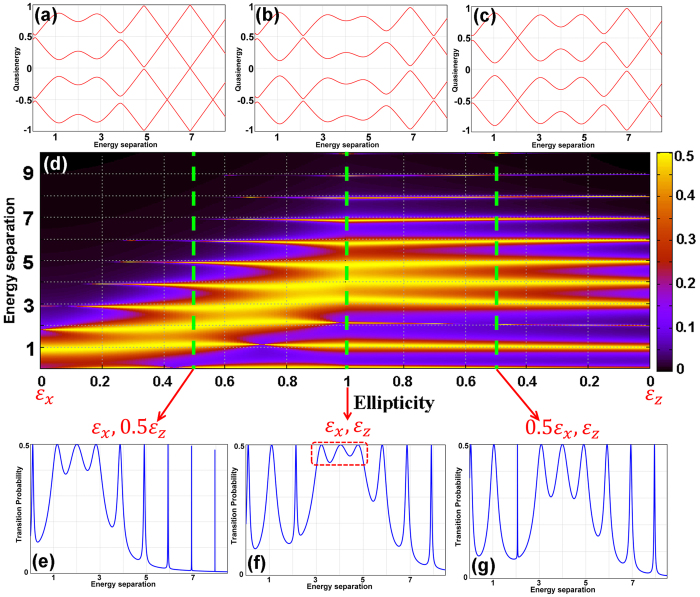
The effect of ellipticity on the multiphoton resonance. (**a**–**c**) The four branches of the quasienergies of the system at the ellipticity values resulting the fields contributions of (**a**) 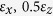, (**b**) 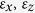, and (**c**) 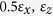. (**d**) The contour plot of the transition probability with respect to the ellipticity parameter and the energy separation between the two states. The dashed green lines indicate the position of the presented three case scenarios. (**e**–**g**) The transition probabilities corresponding to the cases (**a**–**c**).

In Fig. 5, we present the results on how the ellipticity of the field affect the multiphoton resonances between the two states of the qubit. Figure 5(d) is the contour plot of the transition probability with respect to the ellipticity parameter and the energy separation between the two states. The two ends of this plot, where the ellipticity is zero, correspond to the cases when we only have transverse coupling (left end) or only longitudinal coupling (right end). These two extreme cases were examined before separately. At the middle of this plot is the case when the both transverse and longitudinal couplings are fully engaged in the process. Indicated by the dashed red box in Fig. 5(f), as a result of such strong coupling, the three-to-five photon transition probabilities are maximized, compared to the other two case scenarios presented in Fig. 5(e,g). This can also be seen in the corresponding quasienergy plot, Fig. 5(b). The widely-expanded avoided crossing area between the three-to-five photon resonances in this figure indicates that the lower (blue) and upper (red) eigenstates are strongly connected and resonance transitions are well pronounced between the states of the two-level system. For comparison, in Fig. 5(a,c) the quasienergies for the cases with the contributions of 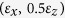 and 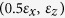 are presented, respectively.

## Conclusion

In summary, we introduced a generalized graph theoretical method to investigate some of the characteristic multiphoton resonance processes and quantum interference phenomena for the superconducting qubit systems driven by intense ac fields. The various interacting designs at arbitrary coupling strengths are modeled by different graph products on colored-weighted graphs. The intuitive picture provided by this beyond rotating-wave approximation approach helps us to demonstrate some characteristic features of the superconducting qubit systems. The population analysis on the virtual dressed states of the product graphs is used to explain the nonlinear phenomenon of multiphoton suppression at longitudinal coupling case. One future step is to extend this method to higher dimensional systems and to study the topological features of their manifolds.

## Additional Information

**How to cite this article**: Jooya, H. Z. *et al*. A graph-theoretical representation of multiphoton resonance processes in superconducting quantum circuits. *Sci. Rep.* **6**, 37544; doi: 10.1038/srep37544 (2016).

**Publisher’s note:** Springer Nature remains neutral with regard to jurisdictional claims in published maps and institutional affiliations.
